# TIPE1 Suppresses Growth and Metastasis of Ovarian Cancer

**DOI:** 10.1155/2021/5538911

**Published:** 2021-06-03

**Authors:** Zhenyu Zhang, Minghui Chang, Xingguo Song, Kangyu Wang, Wenjuan Sun, Hongxin Ma, Xiaohui Yan, Yuhong Sun, Xianrang Song, Li Xie

**Affiliations:** ^1^Department of Clinical Laboratory, Shandong Cancer Hospital and Institute, Shandong First Medical University and Shandong Academy of Medical Sciences, Jinan, Shandong, China; ^2^Department of Obstetrics, The Second Hospital of Shandong University, Jinan, Shandong, China; ^3^Department of Gynecologic Oncology, Shandong Cancer Hospital and Institute, Shandong First Medical University and Shandong Academy of Medical Sciences, Jinan, Shandong, China; ^4^Department of Pathology, Shandong Cancer Hospital and Institute, Shandong First Medical University and Shandong Academy of Medical Sciences, Jinan, Shandong, China; ^5^Shandong Provincial Key Laboratory of Radiation Oncology, Shandong Cancer Hospital and Institute, Shandong First Medical University and Shandong Academy of Medical Sciences, Jinan, Shandong, China

## Abstract

TIPE1, a newly identified member in TIPE (TNFAIP8) family, plays an important role in tumorigenesis and immune regulation, but its role in ovarian cancer, especially in tumor metastasis, remains unknown. In the current study, we aimed to reveal the protein expression spectrum of TIPE1 in normal human tissues and explored its relationship with metastasis in ovarian cancer. The results of IHC staining showed that TIPE1 protein was not only detected in cytoplasm in most human tissues but also expressed in both cytoplasm and nucleus in squamous epithelium and some epithelial-derived cells with secretory functions, such as esophagus, cervix uteri and ovary, and thyroid gland. Moreover, TIPE1 protein was downregulated in ovarian cancer tissues compared with that in the paracancerous. More importantly, TIPE1 suppressed tumorigenesis and metastasis of ovarian cancer *in vitro* and *in vivo*, as evidence shows its ability to suppress growth, colony formation, migration, and epithelial-mesenchymal transition (EMT) of ovarian cancer. Taken together, our results demonstrate the suppressor role of TIPE1 in ovarian cancer metastasis, indicating TIPE1 might be a metastasis predictor and a novel therapeutic target for ovarian cancer.

## 1. Introduction

Ovarian cancer, the most common gynecological malignancy worldwide, has lower incidence than cervical cancer and uterine body cancer; however the death rate is the highest of all gynecological tumors, which seriously poses a threat to women's lives [1]. Despite making unremitting efforts for improving treatment strategies, involving surgery, chemotherapy, and surgery combined with chemotherapy and target therapy in the past forty years, the survival rate had no obvious improvement [2]. Unfortunately, metastasis and recurrence might occur in more than 80% of patients and the 10-year rate of disease-free survival among patients is below 15% [3]. This is largely due to the abnormal growth and increased cell metastasis potential of ovarian cancer cells. Therefore, seeking novel molecular alterations involved in the abnormal cell growth leading to tumor progression would facilitate the realization and development of therapies for ovarian cancer.

TIPE1, a newly identified member of TIPE (tumor necrosis factor-*α*-induced protein 8) family, was first reported to modulate necroptosis and apoptosis in 2008 [4, 5]. Recent studies had suggested that TIPE1 may play a significant role in cancer development and progression. Our previous studies found that TIPE1 was downregulated in hepatocellular carcinoma which could induce caspase-mediated apoptosis and inhibit HCC cell growth both *in vitro* and *in vivo* [6]. Similar results were confirmed and presented in lung cancer [7], gastric cancer [8], osteosarcoma [9], breast cancer [10, 11], and colorectal cancer [12]. A recent study reported that TIPE1 was decreased in tissue microarrays and inhibited proliferation through inducing apoptosis in ovarian cancer cells [13], but whether TIPE1 related to tumor metastasis was not mentioned. Therefore, it is necessary to explore the relationship between TIPE1 and metastasis and to further clarify its anti-tumor effect in ovarian cancer. In addition, TIPE1 seems to act opposite functions in different cancers. Zhao et al. discovered that TIPE1 could promote cervical cancer progression by repression of p53 acetylation [14] and Liu et al. demonstrated that TIPE1 could accelerate NPC cell proliferation by inhibiting autophagy via the AMPK/mTOR signaling pathway [15]. These findings indicated that the functions of TIPE1 may differ in different tissues. Murine TIPE1 was distributed widely in various mouse tissues [16], but the protein profile of human TIPE1 was still unknown. Hence, it is crucial to clarify the protein expression spectrum of TIPE1 in normal human tissues.

In the current study, we investigated the expression and function of TIPE1 in ovarian cancer. The expression of TIPE in clinical specimens and pathological data indicated that TIPE1 was a suppressor gene in ovarian cancer and it was negatively correlated with cancer metastasis. Furthermore, cell biology assays confirmed that overexpression of TIPE1 could inhibit tumor cell growth and migration. In addition, we presented the expression spectrum of TIPE1 protein in normal human tissues and found that TIPE1 protein was not only detected in cytoplasm of most human tissues, but also detected in nucleus of squamous epithelium and epithelial-derived cells, such as esophagus, cervix uteri, ovary, and thyroid gland cells. Our findings revealed the function of TIPE1 in ovarian cancer and suggested that TIPE1 may be a novel therapeutic target for patients.

## 2. Materials and Methods

### 2.1. Clinical Tissue Samples

The normal human tissue microarray was purchased from the Shanghai Outdo Biotech Company (Shanghai, China). Fresh samples including 18 cancer tissues and 10 non-cancer tissues came from ovarian cancer patients who underwent surgery from March 2018 to July 2018 in Shandong Cancer Hospital and Institute. Informed consent was obtained from all patients before the study was initiated with approval of the Shandong Cancer Hospital and Institute Ethics Committee in accordance with the Declaration of Helsinki. A total of 281 ovarian cancer tissues and 89 non-cancer tissues involved in this study contained the following two parts: 24 ovarian cancer pathological sections and 26 non-cancer pathological sections obtained from the Second Hospital of Shandong University: 5 microarrays containing 257 ovarian cancer tissues and 63 non-cancer tissues purchased and carried out IHC experiments from the Shanghai Outdo Biotech Company (Shanghai, China), Guilin Fanpu Biotech Company (Guilin, China), and Wuhan Service Biotech Company (Wuhan, China), and all information of ovarian cancer patients was provided by the companies.

### 2.2. IHC Staining

IHC using the anti-TIPE1 antibody (orb186311, Biorbyt Ltd, Cambridgeshire, UK) was performed according to standard protocols as described previously6. Staining intensity of normal human tissues was scored as follows: no staining = ±; weak staining = +; moderate staining = ++; strong staining = +++. To assess the average degree of staining, multiple regions were analyzed, and at least 100 cells were assessed. The histochemistry score of TIPE1 expression in ovarian cancer tissues and non-cancer tissues was assessed by computer system [17]. The formula for the H-score is as follows: Histoscore = Σ (I × Pi), where I = intensity of staining and Pi = percentage of stained cells, producing a cytoplasmic score ranging from 0 to 300, the higher the score, the stronger the positive staining.

### 2.3. Cell Culture and Transfection

The human ovarian cancer cell lines A2780 and ES2 were purchased from Keygen Biotech (Jiangsu, China). SKOV3 cells were purchased from American Type Culture Collection (ATCC, Manassas, VA, USA). A2780 and ES2 cells were cultured in Dulbecco's modified Eagle's medium (Thermo Fisher, Carlsbad, CA, USA). SKOV3 cells were cultured in McCoy's 5A medium (Thermo Fisher, Carlsbad, CA, USA). All the media were supplemented with 10% fetal bovine serum (Thermo Fisher) and antibiotics (penicillin/streptomycin, 100 U/ml, Beyotime, Beijing, China) at 37°C with 5% CO2. The lentivirus-based TIPE1 overexpression system was purchased from Genechem Company (Shanghai, China). The lenti-TIPE1 was employed to infect A2780, ES2 and SKOV3 cells, and puromycin (2 *μ*g/ml, Beyotime, Beijing, China) was added to select the stable TIPE1-expression cells.

### 2.4. MTT Assay

Cell proliferation analysis was performed by a conventional MTT cell viability assay. Cells were seeded in 96-well plates at density of 2000 per well, and 10 *μ*l of the MTT (5 mg/mL in phosphate-buffered saline) was added per well followed by incubation at 37°C in 5% CO2 for 4 hours before the setting time points. Formazan crystals were solubilized with 100 *μ*l of acidified (0.01 M HCl) 10% sodium dodecyl sulfate (SDS) overnight at 37°C. Absorbance at 570 nm was read on a Bio-Rad 680 microplate reader (Bio-Rad Laboratories, CA, USA).

### 2.5. RNA Extraction and Real-Time RT-PCR

Total RNA was extracted from ovarian cancer cells using Trizol reagent (Invitrogen, Carlsbad, CA, USA) following manufacturer's instructions. After reverse transcription with PrimeScript™ RT reagent Kit (Takara RR047 A, Tokyo, Japan), the complementary DNA was used for real-time PCR with TB Green® Premix Ex Taq™ II (Takara RR820 A, Tokyo, Japan). Relative levels of TIPE1 expression were determined with ACTB as the control. The primer for TIPE1 was as follows: forward primer: 5′'-CAGTGACCTGCTAGATGAG-3'; reverse primer: 5′'-CAAGGTGCTGAGTGAAGT-3'.

### 2.6. Colony Formation Assay

Cells were seeded in 6-well plate at a density of 200 per well and cultured at 37°C in 5% CO2 for 10–14 days. The plate was then fixed with 4% paraformaldehyde for 30 minutes and stained with 0.1% crystal violet for 1 hour. The plate was washed three times with PBS. The numbers of colonies with more than 50 cells were counted.

### 2.7. Cell Migration Assay

The 24-well plate and matched Transwell chambers (Corning, China) were used for cell migration assay. 1 ^*∗*^105 cells in 100 *μ*l serum-free medium were placed into the upper chamber and 700 *μ*l culture medium with 10% FBS was added to the 24 well plate. After incubating for 48h at 37°C in 5% CO2, the chambers were stained with 0.1% crystal violet and photographed.

### 2.8. Wound Healing Assay

Cells were seeded in a 12-well plate at a density of 1 ^*∗*^106 per well. After growing to 90–95% conﬂuence in plate and a wound was made by dragging a plastic pipette tip across the cell surface, the wounds were recorded at 37°C in 5% CO2 for 48h.

### 2.9. Western Blot

ES2 and SKOV3 cells were washed twice with PBS and then lysed with Lysis buffer (Beyotime, Shanghai, China) supplemented with Protease Inhibitor (Beyotime, Shanghai, China), and centrifuged at 4°C for 15 min. A BCA kit (Thermo Fisher Scientific, Waltham, MA, USA) was used to determine the protein concentration. 40 *μ*g quantity of protein was separated on SDS-PAGE and transferred onto PVDF membranes (Millipore, MA, USA). After blocking with 5% nonfat milk for 2h, the membrane was incubated with primary antibodies overnight and washed three times for 10 min with 1×TBST. Next, the membrane was incubated with the corresponding HRP-conjugated secondary antibodies (anti-mouse or anti-rabbit antibodies, Cell Signaling Technology) for 1 h at room temperature. The reaction was visualized using Clarity Western ECL substrate (Bio-Rad, CA, USA) or detected by exposure to a film. The antibodies used included E-Cadherin, N-Cadherin, Slug, Snail, cl-Caspase9, cl-Caspase3 from Cell Signaling Technology; Flag, Caspase3, GAPDH from Proteintech Group.

### 2.10. *In Vivo* Study

Female BALB/*c* mice (4–6 weeks of age) were purchased from Lingchang Biotech Company (Shanghai, China). Animals were fed according to protocols approved by the Institutional Animal Care and Use Committee of Shandong Cancer Hospital and Institute with full respect to the EU Directive 2010/63/EU for animal experimentation. Approximately 1 ^*∗*^107 Lenti-TIPE1 overexpression SKOV3 cells in 100 *μ*l PBS were transplanted subcutaneously into the 10 mice. The lentivirus vector group was used as control. After reaching 100 mm^3^ in size, size of local tumors and mouse body weight were calculated by measuring two perpendicular diameters (length and width) every three days using a caliper, and the volume was calculated according to the following formula: tumor volume (mm3) = 1/2 × (length × square width). Mice were sacrificed after calculating for 19 days and the tumors were isolated and weighed.

### 2.11. Statistical Analysis

GraphPad Prism 6.02 (GraphPad Software, San Diego, CA, USA) was used for data analysis. Student's *t*-test or one-way ANOVA was applied to determine significant differences between groups. The statistical correlation between clinical characteristics of ovarian cancer and TIPE1 expression levels in tissue was analyzed by the unpaired *t*-test or one-way ANOVA test. *p* < 0.05 was considered statistically significant.

## 3. Results

### 3.1. TIPE1 Protein Expression in Normal Human Tissues

Human TIPE1 protein profile is still unclear till now, so we first detected the protein expression of TIPE1 in normal human tissues, such as digestive system organs (Figure S1), respiratory, muscular and nervous system organs (Figure S2), and reproductive system organs (Figure S3). The IHC results showed that TIPE1 protein was not only detected in the cytoplasm of positively stained cells in most human tissues, but also expressed in both cytoplasm and nucleus of squamous epithelium and some epithelial-derived cells with secretory functions, such as esophagus, cervix uteri, ovary, and thyroid gland. The IHC data are summarized in Table 1. Considering the strongest staining of TIPE1 protein expressed in both cytoplasm and nucleus of ovary cells, we did the following research.

### 3.2. TIPE1 Was Significantly Decreased in Ovarian Cancer and Negatively Correlated with Tumor Metastasis

**Figure 1:**
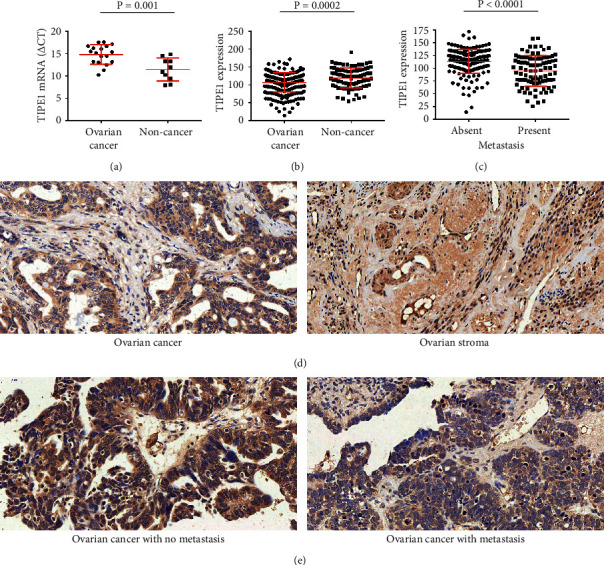
TIPE1 was significantly decreased in ovarian cancer and negatively correlated with tumor metastasis. (a) Relative mRNA levels of TIPE1 in ovarian cancer tissues (*n* = 18) and non-cancer tissues (*n* = 10). (b) Immunohistochemistry score analysis of TIPE1 expression in ovarian cancer tissues (*n* = 281) and non-cancer tissues (*n* = 89). (c) Immunohistochemistry score analysis of TIPE1 expression in metastatic ovarian cancer tissues (*n* = 179) and non-metastatic ovarian cancer tissues (*n* = 102). (d) Representative photographs of TIPE1 IHC staining in ovarian cancer tissues and ovarian stroma tissues. Scale bar = 50 *μ*m. (e) Representative photographs of TIPE1 IHC staining in metastatic ovarian cancer tissues and non-metastatic ovarian cancer tissues. Scale bar = 50 *μ*m.

To illuminate the function of TIPE1 in both physiologic and pathologic conditions in ovarian cancer, we firstly detected the mRNA expression of TIPE1 in ovarian cancer tissues and non-cancer tissues from patients who underwent surgery from March 2018 to July 2018 in Shandong Cancer Hospital and Institute. The results showed that TIPE1 mRNA was lower in cancer tissues (Figure 1(a)). To confirm the above results, we collected ovarian tissue sections and purchased tissue microarrays including 281 cases of ovarian cancer tissues and 89 cases of normal or benign ovarian tissues. IHC staining level of TIPE1 in these tissues indicated that TIPE1-positive cells were rarely observed among ovarian cancer but obvious in the ovarian stroma (Figure 1(d)). In addition, mean IHC score in cancer tissues was significantly lower than those in non-cancer tissues (Figure 1(b)). More importantly, mean IHC score displayed that protein levels of TIPE1 decreased significantly in ovarian cancer tissues characterized by tumor metastasis (Figures 1(c) and 1(e)). Statistical analysis of clinical characteristics of these patients also demonstrated that TIPE1 expression was negatively correlated with metastasis in ovarian cancer (Table 2). Besides, we had also analyzed the relationship between TIPE1 expression and patient age, tumor size, histology, differentiation grade, and clinical stage of 281 ovarian cancer patients, but unfortunately, no statistical significance between them was observed.

### 3.3. TIPE1 Inhibited the Migration of Ovarian Cancer Cells

**Figure 2:**
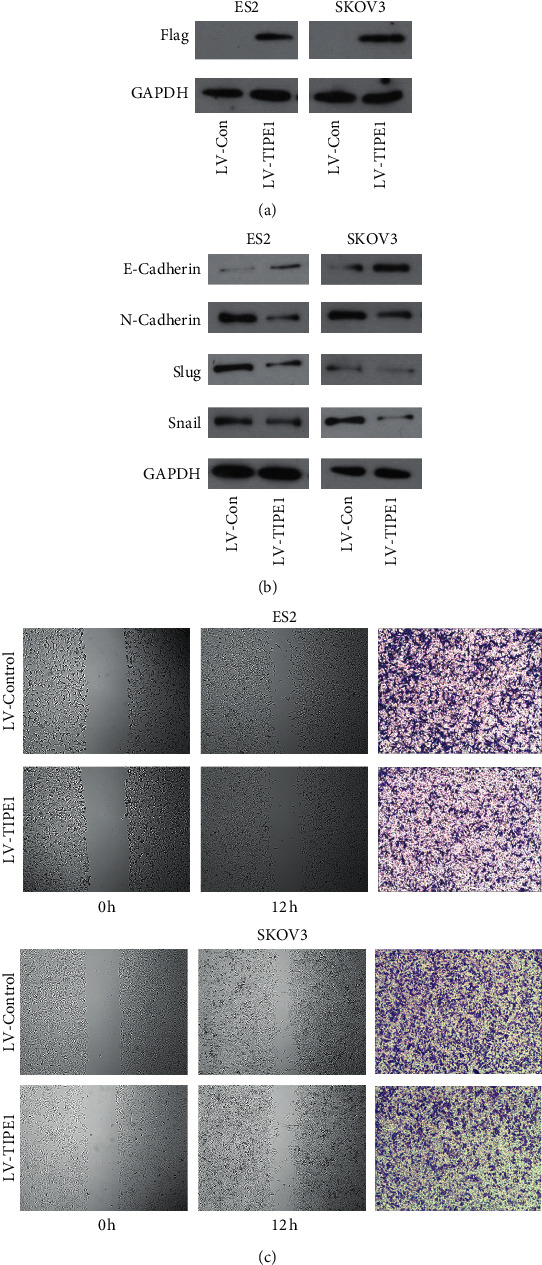
TIPE1 inhibited migration of ovarian cancer cells. (a) The expression of TIPE1 was detected by western blot in ES2 and SKOV3 cells after transfecting lentivirus as indicated. (b) EMT related proteins including E-cadherin, N-cadherin, Slug, and Snail were detected by western blot in ES2 and SKOV3 cells after transfecting lentivirus as indicated. (c) Wound healing assay was performed to determine the migration properties of ES2 and SKOV3 cells. Transwell assay was performed to determine the migration properties of ES2 and SKOV3 cells. Scale bar = 200 *μ*m.

To further determine the function of TIPE1 on the biological behaviors of ovarian cancer cells, we employed lentivirus-based TIPE1 expressing system to infect ovarian cancer cell lines, and puromycin was added to select the stable expression cells. Metastasis is an important factor leading to death and recurrence of ovarian cancer. Our previous studies suggested that TIPE1 was associated with ovarian cancer metastasis, so we attempted to evaluate the effect of TIPE1 on migration ability in ovarian cancer cells. As shown in Figure 2(a), western blot assay confirmed that the overexpressed TIPE1 lentivirus had reliable and well efficiency. Secondly, overexpressed TIPE1 could increase the protein level of E-Cadherin but decrease the protein level of N-Cadherin, Slug, and Snail, indicating that overexpressed TIPE1 could inhibit the epithelial-mesenchymal transition of ovarian cancer cells (Figure 2(b)). Furthermore, overexpression of TIPE1 in ES2 and SKOV3 cells decreased the closure of wound area compared to the control, and Transwell assays also confirmed that TIPE1 down-regulated migratory abilities of ovarian cancer cells (Figure 2(c)).

### 3.4. TIPE1 Suppressed Ovarian Cancer Cell Proliferation *In Vitro*

**Figure 3:**
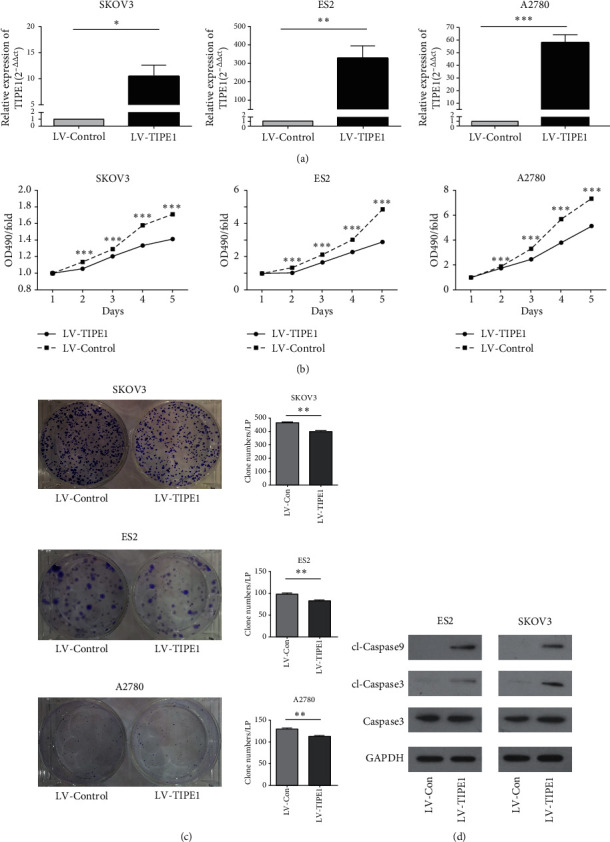
TIPE1 inhibited growth of ovarian cancer cells *in vitro*. (a) Real-time PCR was performed to detected mRNA level of TIPE1 after transfecting lentivirus as indicated. (b) Cell growth curve of A2780, ES2, and SKOV3 cells transfected with TIPE1 and control lentivirus. (c) Colony formation analysis of A2780, ES2, and SKOV3 cells transfected with TIPE1 and control lentivirus. (d) Caspase3, cl-Caspase9, and cl-Caspase3 were detected by western blot in ES2 and SKOV3 cells after transfecting lentivirus as indicated. The results are presented as mean ± SD; ^*∗*^*p* < 0.05, ^*∗∗*^*p* < 0.01, ^*∗∗∗*^*p* < 0.001.

A recent study reported that TIPE1 could inhibit cancer cells by induing apoptosis, and our studies came to the same conclusion. RT-PCR analysis presented the higher expression of TIPE1 mRNA in TIPE1-infected A2780, ES2, and SKOV3 cells (Figure 3(a)). Both MTT assay and colony formation assays were used to estimate the growth abilities of ovarian cancer cells. Overexpression of TIPE1 dramatically decreased cell growth in A2780, ES2 and SKOV3 cells (Figure 3(b)). Colony formation assay also confirmed the inhibiting function of TIPE1 in these cells (Figure 3(c)). Western blot presented that overexpressed TIPE1 increased the expression of cleaved-caspase3 and cleaved-caspase9 in ES2 and SKOV3 cells (Figure 3(d)). These results confirmed the inhibition function of TIPE1 in ovarian cancer cell growth and showed a significant anti-tumor activity of TIPE1.

### 3.5. TIPE1 Suppressed Ovarian Cancer Proliferation *In Vivo*

**Figure 4:**
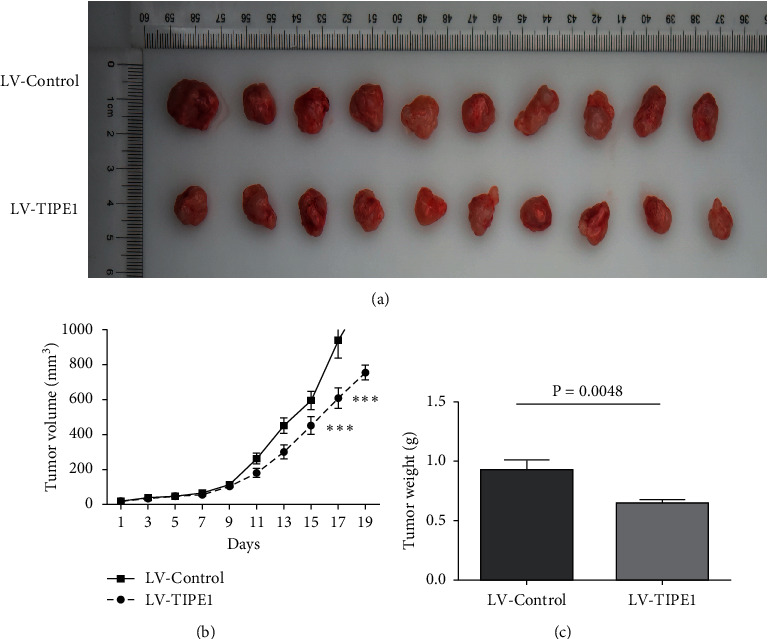
TIPE1 inhibited growth of ovarian cancer *in vivo*. (a) Photograph of tumors in mice that were injected subcutaneously with SKOV3 cells stably transfected with TIPE1 lentivirus or control lentivirus. (b, c) The average tumor weight and tumor volume in each group over a period of 19 days. The results are presented as mean ± SD; ^*∗∗∗*^*p* < 0.001.

Furthermore, we investigated whether TIPE1 arrested tumor xenograft growth *in vivo*. To evaluate the role of TIPE1 in the development of ovarian cancer, we used murine xenograft in Balb/*c* mice with SKOV3 cells with or without TIPE1 overexpression (Figure 4(a)). Tumor growth curve showed that TIPE1 significantly inhibited the growth of SKOV3 xenograft (Figure 4(b)). Consistently, the average tumor weight of lentivirus-TIPE1 group at the time of killing was noticeably less than that of control group (Figure 4(c)). All these data suggested that deficiency of TIPE1 expression was involved in ovarian cancer cells development.

## 4. Discussion

TIPE family (tumor necrosis factor-*α*-induced protein 8) are related to cell death but several members play different roles in tumorigenesis and immune regulation [4, 6, 17–19]. TIPE [20–22] and TIPE3 [18, 23, 24] had been confirmed to possess a carcinogenic effect in tumor development, while TIPE2 [4, 19, 25, 26] was proved to play opposite roles in cell death and tumorigenesis. Unlike the other members, TIPE1 seemed to play more complicated roles in different tumor progressions. We firstly found that TIPE1 was decreased in hepatocelluar carcinoma, increased HCC cell apoptosis, and inhibited cell growth both [6]. Similar results were shown in lung cancer [7], gastric cancer [8], osteosarcoma [9], breast cancer [10, 11], and colorectal cancer [12]. However, some researchers discovered that TIPE1 could promote cell proliferation of cervical cancer and nasopharyngeal carcinoma [15, 27], suggesting that TIPE1 may have different effects in different types of tumors. This phenomenon might be related to the TIPE1 protein expression in different human tissues. However, the protein spectrum of human TIPE1 remains unclear.

In this study, we discovered that TIPE1 protein was universally expressed in the cytoplasm of most human tissues. However, the expression of TIPE1 protein was also detected in both cytoplasm and nucleus in squamous epithelium of skin, esophagus, cervix uteri, and epithelial-derived cells such as breast and thyroid gland. Interestingly, the TIPE1 expression was barely detected in lymphoid tissue of the spleen (Figure 3(e)), but had stronger expression in the plasma cells (Figure 1(g), red arrows) of colonic tissues than those cells around the intestinal tissues. Similarly, the dust cells expressed higher TIPE1 staining in cytoplasm than those cells surrounding the lung tissues (Figure 2(a), right). As we know, plasma cells are derived from B lymphocytes while dust cells originate from macrophages; both of them are activated immune cells. TIPE1 protein is expressed in activated immune cells but not in non-activated immune cells, suggesting that TIPE1 might play a promotional role in immune system. Among all the mentioned human tissues, it should be noted that ovary had the strongest TIPE1 staining, indicating that the function of TIPE1 in ovary might be typical.

Ovarian cancer is a severe gynecological malignancy and it has the highest mortality of all gynecological tumors [1]. Ovarian cancer does not show any obvious symptoms in its early stages; thus most patients are diagnosed at advanced stages accompanied by multiple metastases, limiting the effectiveness of surgery and chemotherapy [14]. These issues might be explained by the particularity of the tumor microenvironment in ovarian. The tumor microenvironment in ovarian cancer consists of stromal cells and extracellular matrix component, reinforced invasion ability of ovarian cancer cells via the complex cross-talk signaling events [28, 29]. Therefore, exploring novel genetic sites would be helpful to understand the tumor development and metastasis mechanism of ovarian cancer.

It was reported that TIPE1 was decreased in cancer tissues and inhibited ovarian cancer cell growth [13], but the relationship of TIPE1 and metastasis was not mentioned. In the current study, we proved that TIPE1 was negatively correlated with metastasis and inhibited the migration ability of cells in ovarian cancer. Moreover, overexpressed TIPE1 increased the protein of E-Cadherin but suppressed N-Cadherin, Slug, and Snail expression, which suggested that TIPE1 could inhibit the EMT of ovarian cancer cells. These findings would help us to understand the complex metastatic ovarian tumor associated microenvironment and provide new insights for the development of tumor targeted agents [29].

## Figures and Tables

**Table 1:** TIPE1 protein expression in normal human tissues.

Tissues	Positive cells	TIPE1 expression	
		Cytoplasm	Nucleus
Esophagus	Stratified squamous epithelium	+++	++
Stomach	Simple columnar epithelium	+	±
	Parietal cells	++	+

Duodenum	Simple columnar epithelium	+	±
Jejunum	Simple columnar epithelium	+	±
Ileum	Simple columnar epithelium	+	±
Appendix	Simple columnar epithelium	+	±
Colon	Simple columnar epithelium	+	±
	Plasma cells	++	±

Rectum	Simple columnar epithelium	+	±
Liver	Hepatocytes	++	±
	Bile canaliculus	+++	±

Pancreas	Islet cells	+++	±
Trachea	Pseudostratified ciliated columnar epithelium	++	++
Lung	Alveolar epithelial cells	+	+
	Dust cells	++	±
Myocardium	Myocardial cells	++	±
Artery wall	Smooth muscle cells	±	±
Skeletal muscle	Skeletal muscle cells	++	±
Telencephalon	Neurons	+	+
Medulla oblongata	Neurons	+	+
Cerebellum	Neurons	±	±
Testis	Spermatogenic epithelium	+	±
Seminal vesicle	Pseudostratified columnar epithelium	++	+++
Prostate	Epithelium	+	±
Cervix uteri	Stratified squamous epithelium	++	++
Endometrium	Uterine gland	+	+
Ovary	Corpus luteum	+++	+++
Skin	Stratified squamous epithelium	++	++
Urinary bladder	Transitional epithelium	++	±
Spleen	Lymphoid tissue	±	±
Thyroid gland	Follicular epithelial cells	+	+
Breast	Acinar cells	++	++

**Table 2:** Characteristics of 281 ovarian cancer patients.

Clinical characteristics	No. of cases	TIPE1 expression (median ± SD)	*P* value
*Age (4 cases missing)*			
<60 years	203	112.4 ± 26.43	0.9617 ^a^
≥60 years	74	111.7 ± 27.22	

*Tumor size (39 cases missing)*			
≤5 cm	55	104.3 ± 30.00	0.0833 ^a^
>5 cm	187	114.2 ± 26.44	

*Histology*			
Serous	186	111.5 ± 27.00	0.5528 ^b^
Mucinous	52	113.5 ± 27.96	
Endometrioid	21	118.0 ± 26.63	
Clear cell	19	113.5 ± 23.29	
Squamous	3	123.8 ± 19.81	

*Metastasis*			
Absent	179	116.1 ± 24.08	<0.0001 ^b^
Present	102	102.2 ± 28.75	

*Differentiation grade*			
Well	42	115.7 ± 24.72	0.2968 ^b^
Moderate	78	110.3 ± 31.83	
Poor	161	112.8 ± 24.6	

*Clinical stage (73 cases missing)*			
Stage I	17	109.1 ± 20.53	0.3252 ^b^
Stage II	45	112.1 ± 30.40	
Stage III	109	112.2 ± 27.48	
Stage IV	37	115.1 ± 20.09	

^a^Unpaired *t*-test (two-tailed). ^b^One-way ANOVA test.

## Data Availability

Data are available upon request to the corresponding author.

## Supplementary Materials

Supplementary Materials Figure S1: TIPE1 protein in digestive system by IHC staining. Figure S2: TIPE1 protein in respiratory system, muscular and nervous system by IHC staining. Figure S3: TIPE1 protein in reproductive system and other tissues by IHC staining.
