# Supplementary material for: TIPE1 Suppresses Growth and Metastasis of Ovarian Cancer
Source: J Oncol. 2021 Jun 3;2021:5538911. doi: 10.1155/2021/5538911 (PMC8195659; doi:10.1155/2021/5538911)
Supplement: Supplementary Materials — Figure S1: TIPE1 protein in digestive system by IHC staining. Figure S2: TIPE1 protein in respiratory system, muscular and nervous system by IHC staining. Figure S3: TIPE1 protein in reproductive system and other tissues by IHC staining. [file 5538911.f1.zip › 5538911.f1/Figure S2.pdf]

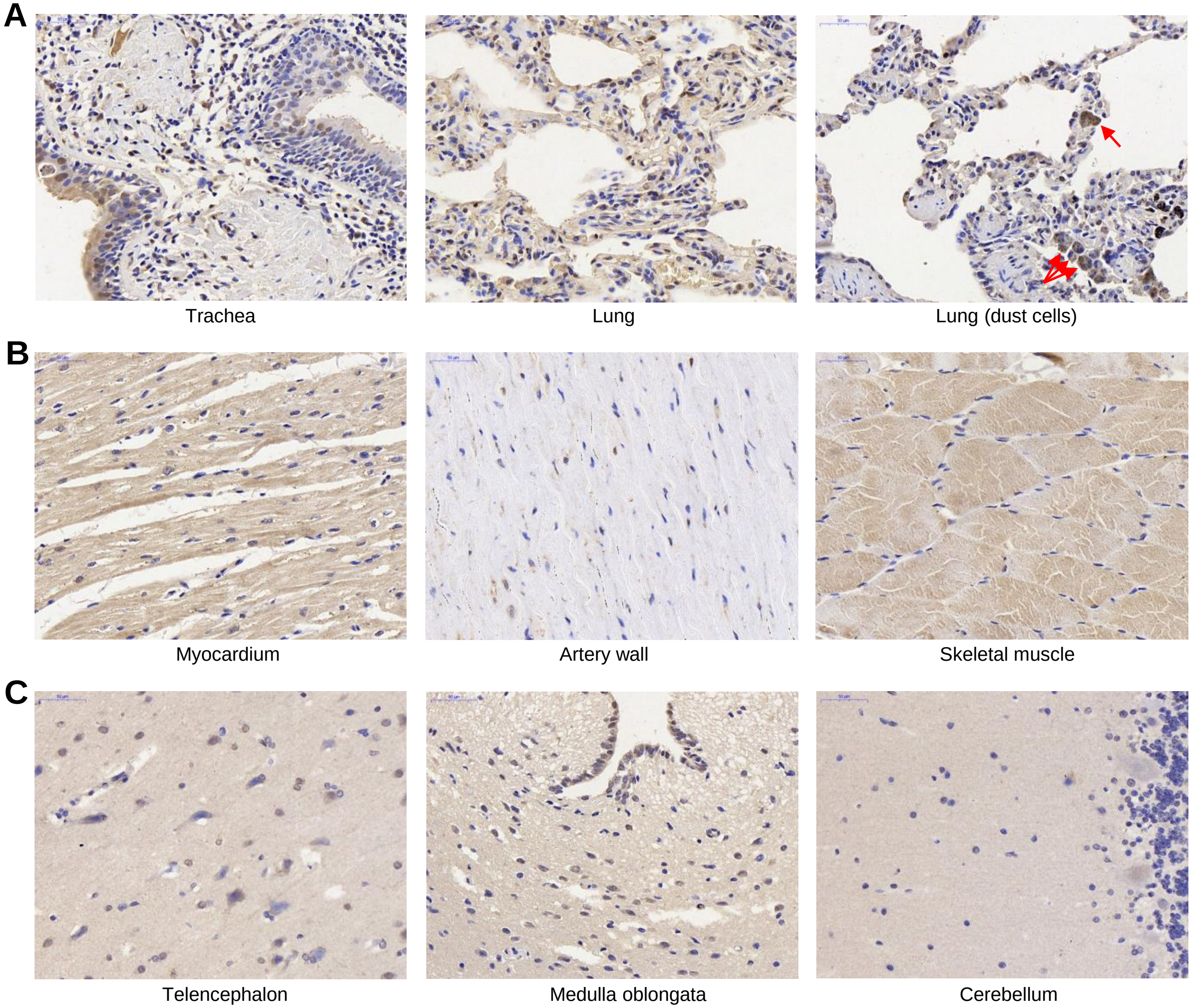
A
Trachea
Lung
Lung (dust cells)
B
Myocardium
Artery wall
Skeletal muscle
C
Telencephalon
Medulla oblongata
Cerebellum

Figure S2
